# Biomechanical Evaluation of Interfragmentary Compression At Tibia Plateau Fractures In Vitro Using Different Fixation Techniques: A CONSORT-Compliant Article: Erratum

**DOI:** 10.1097/01.md.0000464209.93791.98

**Published:** 2015-02-13

In the article “Biomechanical Evaluation of Interfragmentary Compression At Tibia Plateau Fractures In Vitro Using Different Fixation Techniques: A CONSORT-compliant article,^1^ which appeared in Volume 94, Issue 1 of *Medicine*, a line denoting dual authorship was omitted. K. Kojima and B. Gueorguiev contributed equally to the article. The article has since been corrected online.
